# Supplementary material for: Pseudomonas aeruginosa dynamically prioritizes motility and resource recycling during prolonged starvation
Source: mSystems. 2026 Mar 20;11(4):e01439-25. doi: 10.1128/msystems.01439-25 (PMC13098243; doi:10.1128/msystems.01439-25)
Supplement: Supplemental material — Figures S1-S5 and additional methodological details. [file msystems.01439-25-s0002.pdf]

Supplemental Information:

1. Supplemental figures S1-S5
2. Supplemental materials and methods

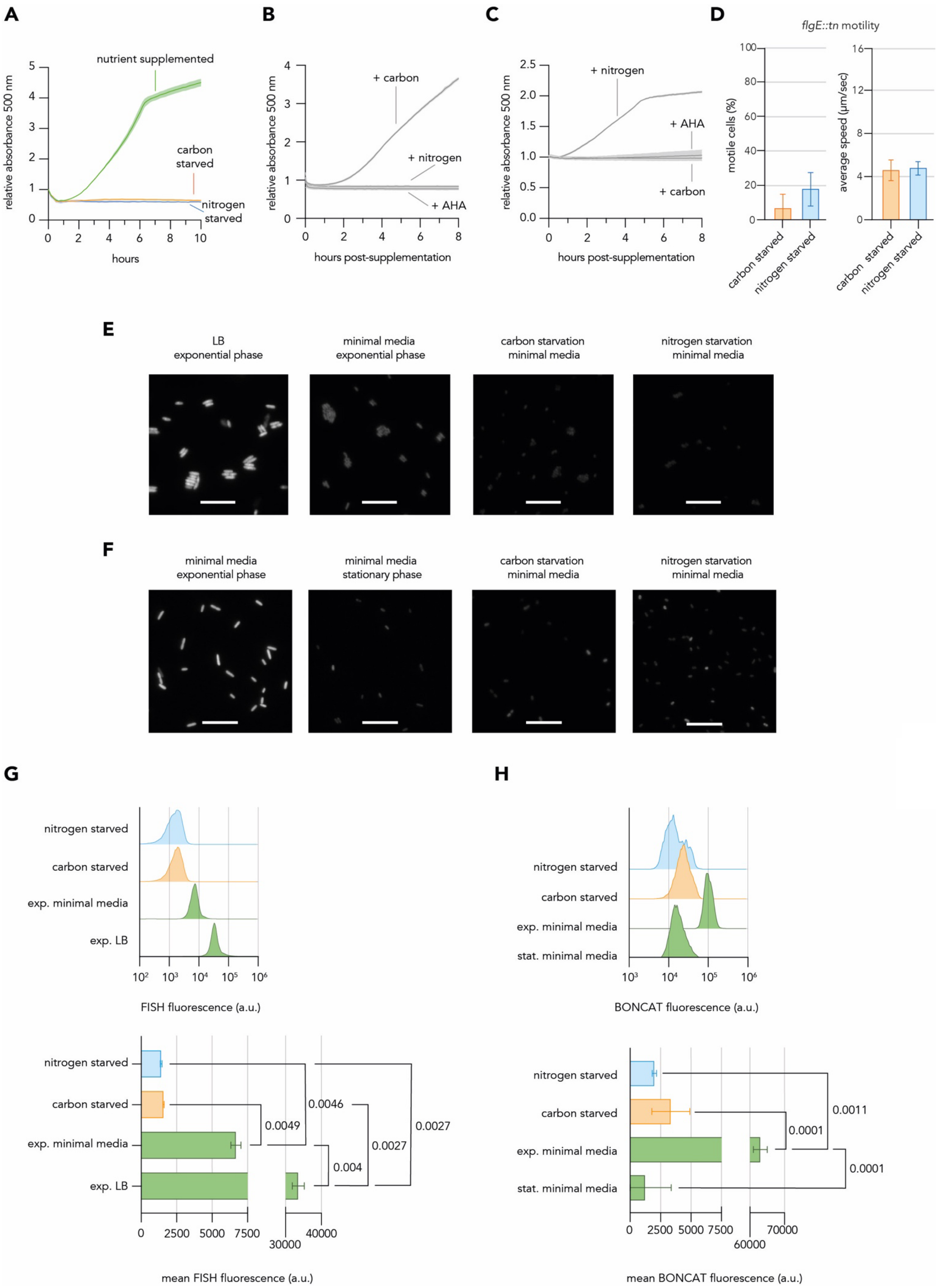

Supplemental Figure S1

Line graphs (**A**) show the optical density changes during the first 10 hours of response of *P. aeruginosa* LB overnight cultures to resuspension in either nutrient-supplemented minimal media; carbon-starvation minimal media; or

nitrogen starvation minimal media, measured every six minutes in a plate reader. While all three conditions show an initial small decrease in optical density, no substantial further changes indicative of growth are seen in either starvation condition. Resuscitation line graphs show changes in optical density in the response of 48-hour carbon starved (**B**) and 48-hour nitrogen starved (**C**) *P. aeruginosa* cultures to supplementation with a carbon source (45 mM sodium succinate), a nitrogen source (15 mM ammonium chloride), or the amino acid analogue azido-homo-alanine (200 µM AHA). Supplementation with the missing nutrient stimulates growth, confirming that this nutrient is still limiting. Fraction of motile cells and average speeds of motile cells are shown for a flagellar mutant transposon mutant, *flgE::tn,* to demonstrate that motility detected during starvation in the wild type can be substantially attributed to flagellar motility (**D**). Representative fluorescence microscopy images show *P. aeruginosa* cultures which were fixed during growth-permitting conditions or following 50 hours of carbon or nitrogen starvation conditions and which were FISH-stained using a ribosome-directed probe (**E**) or BONCAT-labelled for 30 minutes with 500 µM AHA (**F**). In microscopy images, the scale bar represents 10 µm. Histograms and bar graphs show cellular fluorescence values and the mean cellular fluorescence values from *P. aeruginosa* cultures which were fixed during growth-permitting or starvation conditions and which were FISH-stained using a ribosome-directed probe (**G**) or BONCAT-labelled for 30 minutes with 500 µM AHA (**H**). All data represent the mean value calculated from three biological replicates with standard deviation displayed as error bars. Histograms show data from a single representative biological replicate. One-way analysis of variance was conducted to assess differences among conditions, with Dunnett's T3 multiple comparisons test used to calculate adjusted p-values for pairwise comparisons and significant values shown. a.u. = arbitrary units.

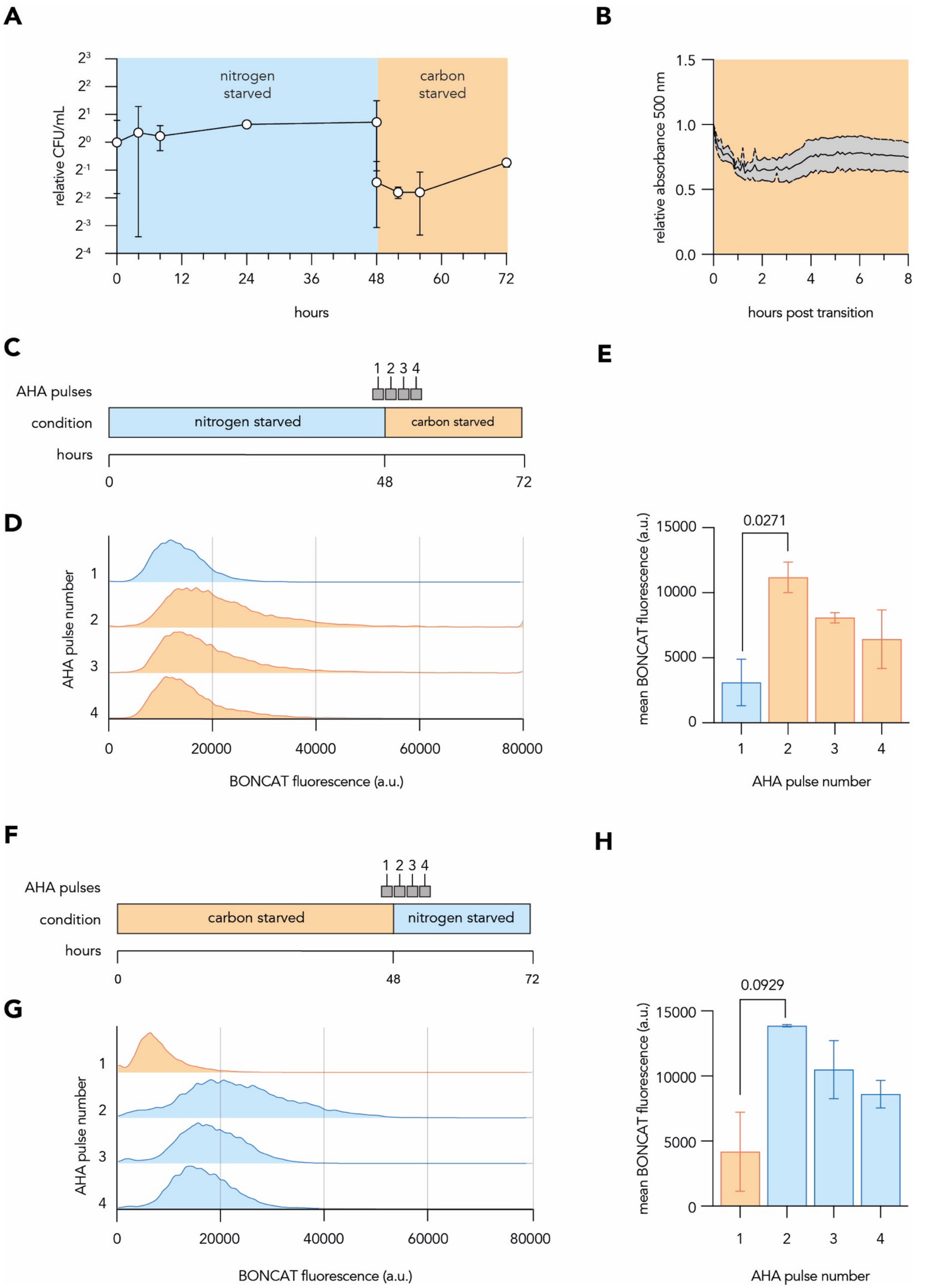

Supplemental Figure S2

Line graphs show the trends in colony-forming units (**A**) and absorbance 500nm (**B**) during the rapid transition of *P. aeruginosa* cultures between nitrogen and carbon starvation minimal media. Schematics show the time-course and

accompanying two-hour 500 µM AHA pulses applied to *P. aeruginosa* cultures during the BONCAT labelling of a transition from nitrogen starvation to carbon starvation minimal media (**C**) and the transition from carbon starvation to nitrogen starvation minimal media (**F**). Histograms (**D** and **G**) show representative distributions of cellular fluorescence returned from each two-hour AHA pulse applied to a single replicate of transitioning cultures. Bar graphs (**E** and **H**) show the average of mean fluorescence values from each BONCAT labelling pulse applied to 3 replicate transitioning cultures. All data represent the mean values calculated from three biological replicates with error bars displaying standard deviation. One-way analysis of variance was conducted to assess differences in BONCAT signal across the labelling periods, with Dunnett's T3 multiple comparisons test used to calculate adjusted p-values for pairwise comparisons and selected values of interest shown. a.u. = arbitrary units.

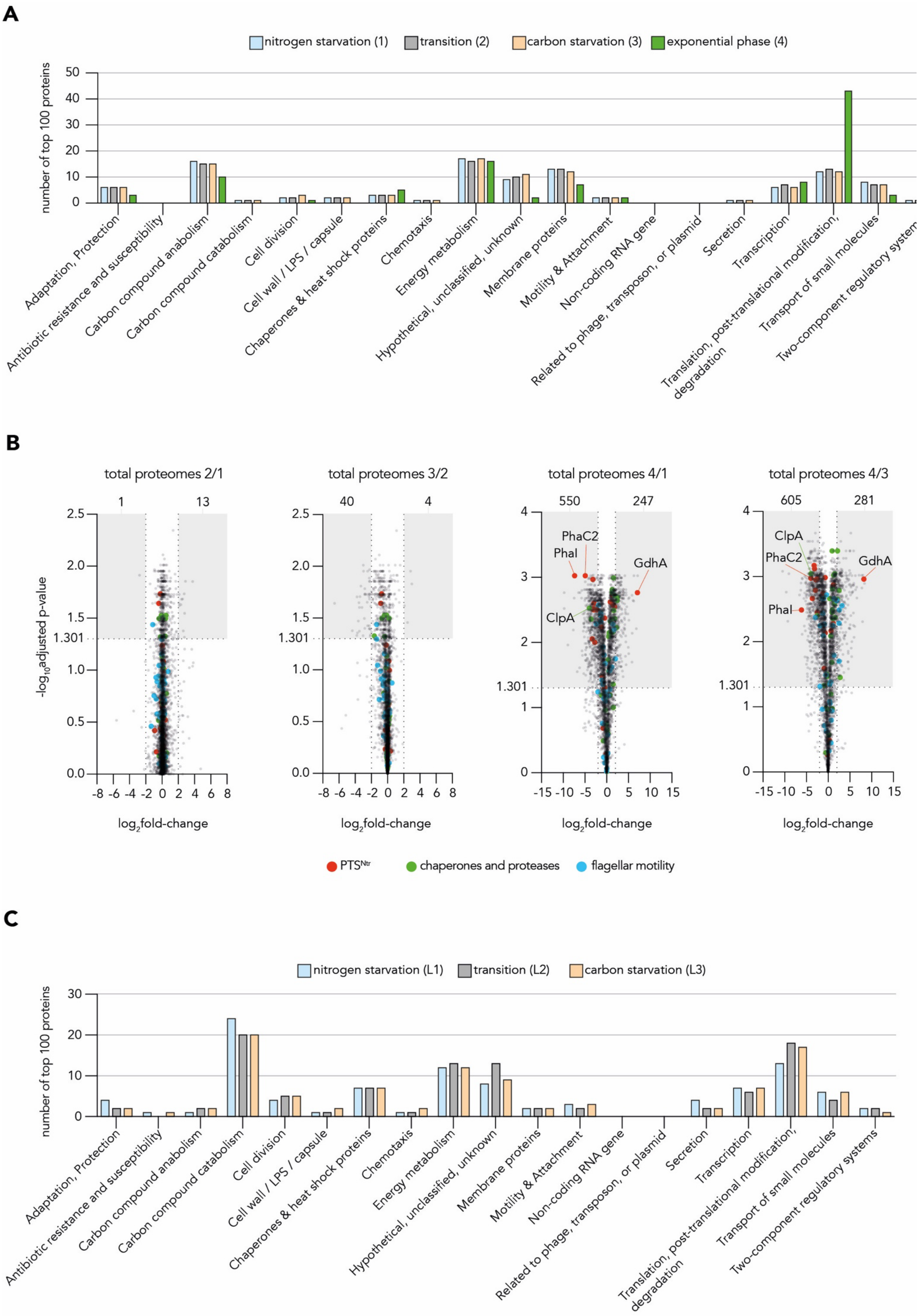

Supplemental Figure S3

Bar graphs (**A**) show the occupancy of modified PseudoCAP groups by the top hundred most abundant proteins retrieved from the sampling timepoints of label-free proteomic experiments. Volcano plots (**B**) show the fold-change in abundance of proteins when comparing the total proteomes retrieved from different sampling periods of the label-free proteomic experiment. In each volcano plot, shaded boxes highlight the nascent proteins which passed fold-change (log_2foldchange > 2 or < -2) and adjusted p-value (log_{10}adjusted-p-value > 1.301) cut-offs to be considered as robust changes in abundance between compared proteomes, with the number of such proteins denoted above each shaded box. The larger coloured dots highlight individual proteins belonging to key physiological functions. Bar graphs (**C**) show the occupancy of modified PseudoCAP groups by the top hundred most abundant proteins retrieved from the labelling periods of BONCAT proteomic experiments outlined in Fig. 3D. All analyses used mean abundances calculated from four biological replicates.

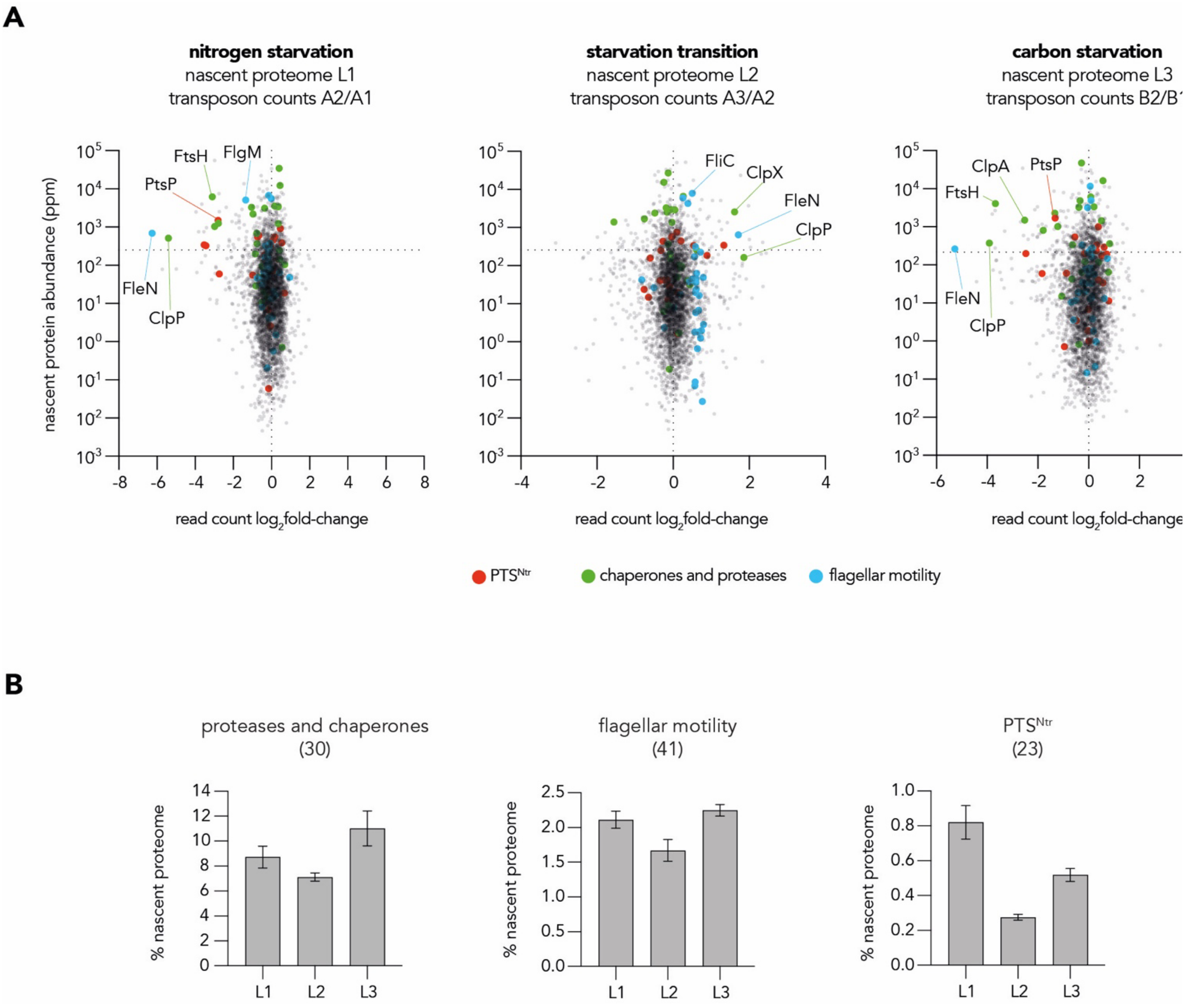

Supplemental Figure S4

Scatter plots (**A**) combine BONCAT proteomic (outlined in Fig. 3D) and transposon-insertion sequencing data (outlined in Fig. 4A) and show the normalised protein abundance and transposon read count of individual proteins/genes detected in both datasets under comparable conditions. In each plot, a dotted line crosses the y-axis at values corresponding to the mean abundance of all normalised proteins within the refined proteomic dataset, and dotted line crosses the x-axis at x=0. The larger coloured dots highlight individual proteins belonging to key physiological features. Bar graphs (**B**) show the percentage occupancy of nascent proteomes by nascent proteins belonging to three key physiological features at each labelling period used in BONCAT proteomic experiments. The number of genes each discrete cohort is composed of are noted in brackets. All analyses used mean abundances calculated from four biological replicates of BONCAT proteomic data and mean read counts calculated from six biological replicates of TnSeq data. Bar graphs used the mean value calculated from four biological replicates, with error bars displaying standard deviation.

A

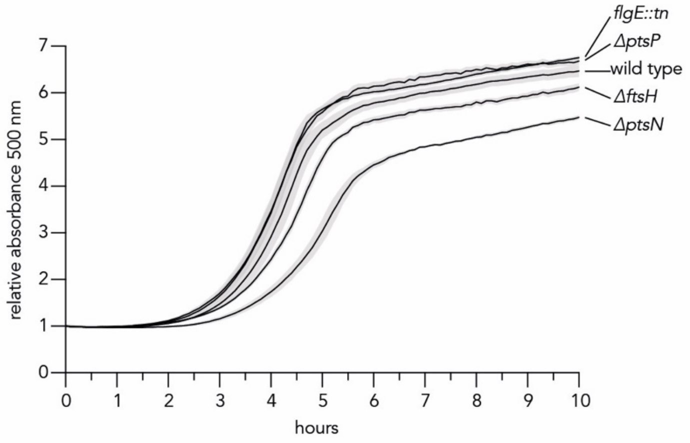

B

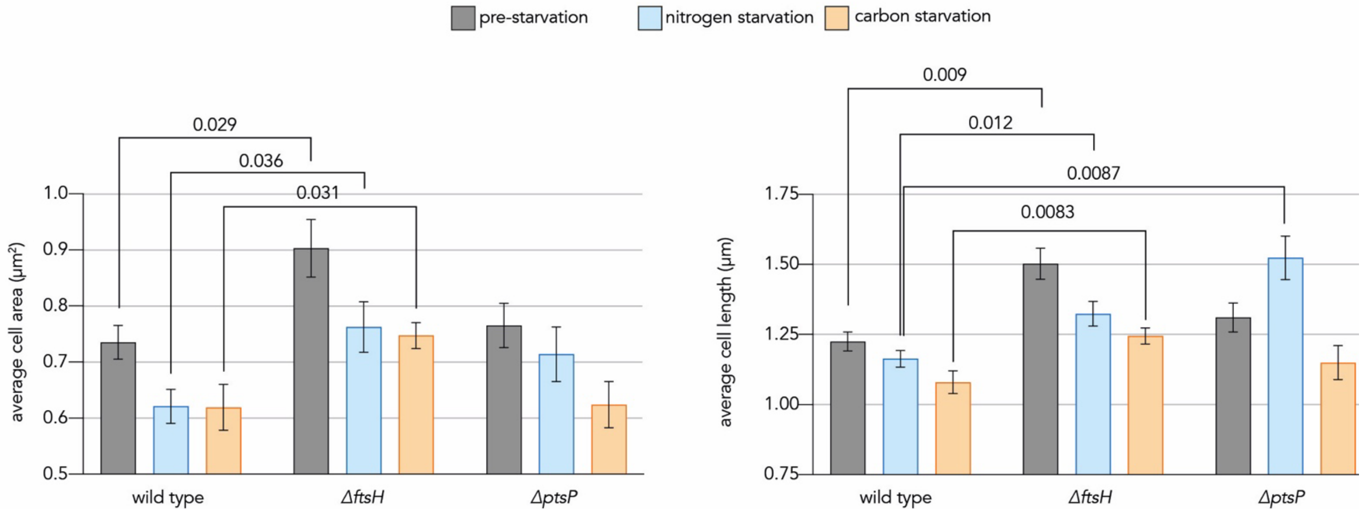

Supplemental Figure S5

Line graph (**A**) shows the growth of mutant *P. aeruginosa* strains in LB media from a starting OD 0.01 as measured by absorbance at 500nm (n=9). Bar graphs (**B**) show the average cell area and cell length of fixed *P. aeruginosa* cells of different strains in LB stationary phase before resuspension in starvation media and following 48-hour incubation in nitrogen or carbon starvation media. All data represent the mean value calculated from three biological replicates with standard deviation displayed as error bars. One-way analysis of variance was conducted to compare the cell areas (left) or lengths (right) among the three strains within each of the three conditions and Dunnett's T3 multiple comparisons test was used to calculate adjusted p-values for pairwise comparisons of each mutant to the WT in each condition. Only significant differences from the wild type are annotated. a.u. = arbitrary units.

Supplemental Materials and Methods:

Strain list

Genotypes and absence of background mutations were confirmed for all *P. aeruginosa* strains by whole-genome sequencing.

Organism	Strain ID	Strain Name	Genotype/Description	Origin
Pseudomonas aeruginosa UCBPP-PA14	MB001	PA14 WT	wild type strain	Diane K Newman lab[1]
	-	PA14 *flgE*::tn	*MAR2xT7* insertion within *flgE* (*PA14_50450*)	Ausubel lab ordered transposon library[2]
	MB098	PA14 Δ*ptsN*	clean deletion of *ptsN* (*PA14_57960*)	This study
	MB057	PA14 Δ*ptsP*	clean deletion of *ptsP* (*PA14_04410*)	Dianne K Newman lab[3]
	MB301	PA14 Δ*ftsH*	clean deletion of *ftsH* (*PA14_62860*)	Dianne K Newman lab[3]
	MB096	PA14 P_{rpsG}-mScarlet	mScarlet under control of P_{rpsG} introduced chromosomally at Tn7 site	This study
Escherichia coli	MB012	SM10(λ*pir)* pIT2	Harbours pIT2 plasmid used for transposon mutant library construction	Dianne K Newman lab[3]
	MB095	DH5α pUC18T-miniTn7T- Gm^R-P_{rpsG}-mScarlet	Harbours pUC18T-miniTn7T delivery plasmid for integrating cassettes into Tn7 site	This study
	MB097	DH5α pMQ30-*ptsN* del	Harbours pMQ30 suicide vector for clean deletion of *ptsN* gene[4]	This study
	MB010	HB101 pRK2013	Helper strain carrying machinery for conjugation into *P. aeruginosa*	Schweizer lab[5]
	MB011	SM10(λ*pir)* pTNS2	Helper strain carrying site-specific recombinase for integration into Tn7 site	Schweizer lab[5]

Primer list

Name	Sequence	purpose
ptsN_US_F	TGGGTAACGCCAGGGTTTTCCCAGTCACGACGTTGTA AAAGGCGGGGAATGCTCGTCCAC	clean deletion of ptsN
ptsN_US_R	TGACGATGATCAGGCGCATGAGGGGAGGGGAGATCA GCGG	clean deletion of ptsN
ptsN_DS_F	CCGCTGATCTCCCCTCCCCTCATGCGCCTGATCATC GTCA	clean deletion of ptsN
ptsN_DS_R	GTGAGCGGATAACAATTTCACACAGGAAACAGCTATG ACAACTCGGGCTTCCAGTAGGGG	clean deletion of ptsN
pMQ30 F	TTTATGCTTCCGGCTCGTAT	amplification of pMQ30
pMQ30 R	GTGCTGCAAGGCGATTAAGTT	amplification of pMQ30
rpsG_mScarlet_F	GACCGTTTGAGGGCTTATCAatggtgagcaagggcgaggc	generation of construct for constitutive mScarlet expression
rpsG_mScarlet_R	TGATGGCAGGATCAGCGAttacttgtacagctcgtccat	generation of construct for constitutive mScarlet expression
rpsG_mScarlet_pF	atggacgagctgtacaagtaaTCGCTGATCCTGCCATCA	generation of construct for constitutive mScarlet expression
rpsG_mScarlet_pR	gcctcgcccttgctcaccatTGATAAGCCCTCAAACGGTC	generation of construct for constitutive mScarlet expression
TnSeq_Rnd1_F	CTGGATGGAAAACGGGAAAGGTTCCGTCCA	TnSeq sample preparation
TnSeq_Rnd1_R	GTGACTGGAGTTCAGACGTGTGCTCTTCCGATCTGGG GGGGGGGGGGGG	TnSeq sample preparation
TnSeq_Rnd2_F	AATGATACGGCGACCACCGAGATCTACACTCTTTCCC TACACGACGCTCTTCCGATCT	TnSeq sample preparation
TnSeq_Rnd2_R	NEB Next multiplex oligos	TnSeq sample preparation

Strain construction

An unmarked *ptsN* deletion strain *(*Δ*ptsN*) was produced by cloning two 600 bp sequences, encoding sequences upstream and downstream *ptsN* into the pMQ30 suicide vector[4]. These upstream and downstream regions were amplified from *P. aeruginosa* genomic DNA using primers containing additional sequences complementary to those of pMQ30. Gibson assembly was used to combine linearized pMQ30 plasmid (produced by PCR) with the amplified upstream and downstream fragments. Assembly product was used to transform competent *E. coli* DH5α cells, and successful transformants were selected on LB agar containing 100μg/mL gentamycin. The plasmid sequence of a successful transformant was confirmed by Sanger sequencing (MRC PPU DNA Sequencing and Services unit). Following confirmation, the pMQ30-*ptsN*del plasmid was introduced into *P. aeruginosa* UCBPP-PA14 by triparental conjugation. Successful exoconjugants were selected on VBMM medium (3 g/L trisodium citrate, 2 g/L citric acid, 10 g/L K2HPO4,3.5 g/LNaNH4PO4,1mM MgSO4, 100 μMCaCL2,pH 7) containing 100 μg/mL gentamicin as previously described[5] and were then subjected to counterselection on LB plates lacking NaCl and containing 10% (wt/vol) sucrose. Colonies resulting

from homologous re- combination to remove the wild-type copy of *ptsN* and retain the clean deletion were identified by PCR.

For the construction of the strain expressing mScarlet under the control of the *rpsG* promoter, the mScarlet-I encoding sequence was amplified from the pMRE-Tn7-145 plasmid[6] and used to replace the sfGFP ecoding sequence in the plasmid from strain DKN1642[7], by Gibson assembly. The mScarlet-ecoding cassette was integrated into the Tn7 site of the chromosome of WT PA14 using tetraparental conjugation, as described above but with the addition of the pTNS1-carrying helper strain[5].

Transposon-Insertion Sequencing – Library Preparation

The randomly inserting Tn5-based transposon delivery plasmid pIT2[8] was conjugated into UCBPP-PA14 to produce a diverse transposon mutant library. UCBPP-PA14 and *E. coli* SM10(*λpir)* carrying the transposon-bearing pIT2 plasmid were streaked onto LB agar plates and LB + 100 µg/mL carbenicillin agar plates respectively. Following overnight incubation at 37 ℃, cells were scraped from each plate and resuspended in 1 mL of LB. Each suspension was then pelleted and washed once in LB before being resuspended in LB to an optical density of 50 and 100 OD units for PA14 and SM10(*λpir)*/pIT2 respectively. Aliquots (100µL) of each dense culture were then mixed and 50 µL of this mixture was spotted onto LB agar plates and incubated at 37 ℃ for 2.5 hours. Spots were then scraped and resuspended in 16 mL of LB before 100 µL aliquots were spread onto 160 individual LB + 60 µg/mL tetracycline + 10 µg/mL chloramphenicol agar plates. Plates were incubated at 37 ℃ for 24 hours to select for transposon-integrated PA14. The roughly 180,000 resultant colonies were scraped and collected from the plates and made to an OD of ~5 in LB. Glycerol was added to the pooled transposon mutant library which was then aliquoted and stored at - 70 ℃ until its application in subsequent experiments.

Absorbance Readings

The absorbance of cultures at 500 nm was recorded using a Tecan Spark plate reader. At desired timepoints, aliquots of culture were placed in wells of clear-bottom 96-well plates and absorbance was recorded. In resuscitation assays, resuscitation was performed following prolonged incubation of cultures under starvation conditions. Samples (180µL) from aged cultures were aliquoted into wells of a flat-bottomed clear 96-well plate. Concentrated solutions of carbon, nitrogen or carbon + nitrogen were then added to wells to give a final volume of 200µL and a final concentration of 45 mM sodium succinate and/or 30 mM ammonium chloride. In experiments where azidohomoalanine was provided to cultures, the analogue was added to a final concentration of 500 µM. In resuscitation assays and

growth curves, plates were sealed with a BreatheEasy gas-permeable sealing membrane (Diversified Biotech) and placed in a Tecan Spark plate reader pre-warmed to 37 ℃ and set to continuously shake. In all longitudinal experiments, absorbance readings were taken every 6 – 10 minutes.

Agarose Pad Preparation for Microscopy

For each agarose pad, a 65 µL gene-frame (ThermoFisher) was attached to the surface of a standard 25 mm x 75 mm microscopy slide and used as an agarose holder. A 1% agarose solution was melted and ~90 µL was added to a corner of the gene-frame. Immediately, a second slide was pressed on top of the gene-frame, forcing the agarose to completely fill the gene-frame. Gene-frame sandwiches were left to cool and solidify. The upper slide was then slid from the sandwich to reveal the solidified agarose. Sample droplets (0.5 µL) were spotted onto the agarose and allowed to dry before the gene-frame's upper coating was removed and a cover slip was annealed to the exposed adhesive. Agarose pads were always imaged within a week of preparation and were kept at 4 ˚ C in the dark.

Cellular Displacement Analyses of Bacterial Motility

For starvation motility experiments, triplicate cultures of PA14 WT and PA14 *flgE*::tn were grown for 24 hours at 37 ℃ shaking in LB media before being washed and resuspended in nitrogen starvation or carbon starvation MOPS media at an OD of 0.2. Following 48 hours of incubation at 37 ˚ C, 2 µl samples were taken from cultures and spotted onto the centre of a gene frame (ThermoFisher) attached to a glass slide. A coverslip was then adhered to the top of the gene frame, squashing the spot. Focusing at the surface of the coverslip, samples were immediately filmed using the phase-contrast channel of a Nikon Eclipse Ti2 microscope. All films were 15 seconds in length and contained 46 frames (3 fps). The ImageJ plugin TrackMate was used to analyse phase contrast films by identifying individual cells and tracking their displacement between frames of the film[9]. Briefly, the phase contrast images were inverted such that cells were light and the background was dark, and the "thresholding" detection method was used to identify cells in each frame. Identified cells were filtered using an automatic "quality" threshold. Tracks of individual cells across the frames were calculated using the default "Simple LAP Tracker" with "Linking max distance" and "gap-closing max distance" both set to 15 microns, and "gap-closing max frame gap" set to 2. For each film, tracks lasting less than 1.5 seconds were discarded. Cells were considered motile if their total track displacement was more than 4 microns. The mean speeds calculated for each cell in these motile populations were averaged to give the population average speeds displayed. For motility experiments in rich media (LB), overnight stationary phase LB cultures were diluted 1:100 in fresh LB and grown to early exponential phase

(OD=0.2) before spotting 10 μl culture directly onto a slide and imaging as described above. Image processing was the same.

Transition Competition Assay

Cultures of PA14 P_{rpsG}-mScralet and non-labelled strains of interest were grown for 24 hours at 37 ̊C shaking in LB media before being washed and resuspended in nitrogen starvation media at an OD of 0.2. Non-labelled competitor cultures, including a control of PA14 WT, were mixed in equal ratio with PA14 P_{rpsG}-mScatlet and incubated at 37 ̊C shaking. Mixed cultures were incubated for 48 hours in nitrogen starvation MOPS minimal media before being transitioned to carbon starvation MOPS minimal media. Following another 48 hours of incubation, cultures were transitioned back to nitrogen starvation MOPS media. One more transition was applied, into carbon starvation MOPS minimal media, meaning that cultures had experienced a total of three starvation transitions. At the beginning and end of the time-course, small samples of these mixed cultures were taken for inspection via fluorescence microscopy. Fluorescence images of non-labelled and mScarlet-labelled cells were captured, and microbeJ[10] was used to identify and quantify the number of each strain within the mixed cultures. The proportion of either strain within the mixture was calculated at the beginning and end of the time-course. The change in ratio of non-labelled mutant cells at the end of the time-course was then calculated relative to the ratio at the start of the time-course. For each mutant, this "competitive index" was then normalised to the value calculated in control competitions using non-labelled PA14 WT, which exhibited a small but consistent defect when competed against PA14 P_{rpsG}-mScarlet. For each of three biological replicates used in competitions, quantification used images which contained between 90 – 400 cells.

Microscopy and Image Analyses

All images and videos were acquired using a Nikon Eclipse Ti2 microscope fitted with a 60X phase contrast objective (Nikon - MRD31605); a TIPA slide adaptor (Oko Labs); a Nexus optical breadboard (Thorlabs); a camera (Teledyne 48 photometrics - SN A21b204004) and Spectra light engine (Lumencor - 80 10039). Fluorescence of interest was recorded with the following settings; BODIPY (excitation 470 nm LED, emission 510 nm filter cube); AlexaFluor-488 (excitation 470 nm LED, emission 510 nm filter cube); DAPI (excitation 395 nm LED, emission 435 nm filter cube); Cy5 (excitation 640 nm LED, emission 700 nm filter cube) and mScarlet (excitation 575 nm LED, emission 700 nm filter cube). Consistent phase-contrast settings were used across all relevant experiments, while fluorescence settings were calibrated to the brightest sample within each experiment.

All microscopy images were processed in ImageJ. Fluorescent channels were aligned to that of the phase using the ImageJ macro Align_Fluor_Channels, written by Norbert Vischer in 2017. The ImageJ plugin MicrobeJ was then used to identify and segregate individual cells and to quantify average cell shape parameters or average cellular fluorescence for BONCAT, FISH or BODIPY investigations[10]. Microscopy-based experiments which quantified cell area parameters involved data from 60-300 cells per biological replicate.

Flow Cytometry Data Analyses

Fixed samples were analysed on a Novocyte (Agilent) flow cytometer. Forward scatter pulse height (FSC-H) and side scatter height (SSC-H) parameters with logarithmic amplification were used to distinguish fixed bacterial cells from dust and other particulates, as was DAPI staining. Pulse height measurements were used for all fluorescence parameters. BODIPY and AlexaFluor-488 fluorescence was detected using 488 nm excitation and emission was collected at 530/30 nm. DAPI fluorescence was measured using 405 nm excitation and emission was collected at 445/45 nm. Cy5 fluorescence was detected using 640 nm excitation and emission was collected at 675/30. Cytometry data was imported and analysed in FlowJo V10.8.1 (Becton Dickinson). Populations were first gated based on forward-scatter and side-scatter profiles to remove particulate before DNA-containing singlets were isolated by gating for DAPI profile. The resultant gated populations were used to produce fluorescence histograms in FlowJo and Adobe Illustrator, with the mean value of these histograms being directly extracted from FlowJo to provide and compare “average” fluorescent values of samples. Quantification of population average BODIPY, BONCAT and FISH fluorescence made use of between 7,000-40,000 cells per biological replicate.

Modification of PseudoCAP and Choice of Key Features

For ease of data analyses and interpretation, established functional PseudoCAP[11] were modified (Supplementary file 1). In cases where individual proteins had numerous PseudoCAP annotations, the first annotation was used. In cases where the first annotation was “hypothetical, unclassified, unknown” or “putative enzyme”, the second annotation was used. Several PseudoCAP categories were merged to simplify interpretation of data. The categories “amino acid biosynthesis and metabolism”; “biosynthesis of cofactors, prosthetic groups and carriers”; “central intermediary metabolism”; “fatty acid and phospholipid metabolism” and “nucleotide biogenesis and metabolism” were merged into “carbon compound anabolism”. The categories “cell division” and “DNA replication, recombination, modification and repair” were merged into “cell division”. The categories “protein secretion/export apparatus” and “secreted factors (toxins, enzymes, alginate) were merged into

"secretion". The categories "transcription, RNA processing and degradation" and "transcriptional regulators" were merged into "transcription".

The key physiological functions we focused on (flagellar motility; proteases and chaperones and the phospho-transferase system) were defined and populated by selecting genes/proteins relevant to each (Supplemental file 1). For flagellar motility, members of the modified PseudoCAP category "motility & attachment" were largely used. Additional genes known to encode structural and regulatory flagellar components were also included. For proteases and chaperones, members of the modified PseudoCAP category "chaperones & heat shock proteins" were largely used. Additional genes known to encode proteases were also included. For the phospho-transferase system, genes encoding known components of the nitrogen-sensing and fructose-sensing phospho-transferase systems of *Pseudomonas aeruginosa* were included. Genes encoding metabolic enzymes whose products are predicted to influence these systems[12,13] were also included. Genes relevant to the production of polyhydroxyalkanoate; an intracellular carbon storage device which is known to be influenced by phospho-transferase systems in *Pseudomonas putida*[14] were also included.

Total proteomics – Data Analysis

Data retrieved from total proteomic experiments were largely handled in Microsoft Excel and was assisted by described processing methodology[15]. For each sample, individual protein abundance was converted to parts per million to normalise for differences in processing and loading. The resulting values were considered reflective of the fraction of the total proteome represented by each protein, with non-detected proteins having an abundance set to 0. A principal components analysis of all samples was performed using the online tool ClustVis[16]. Refined data was uploaded to ClustVis, which returned numerous component values for each sample. For each sample, the first two components (PC1 and PC2) were extracted and plotted to confirm the clustering of replicates. In further analyses, the average abundance calculated from four biological replicates of each sampling timepoint was used. For abundance analyses, the top 100 most abundant proteins from each averaged sample timepoint were functionally classified into modified PseudoCAP (Supplemental file 1). Bar graphs of category occupancy were then produced for each sampling time point. For volcano plots, the fold-change for each detected protein was calculated using the average protein abundance for each individual sampling timepoint. Two tailed paired t-tests were used to calculate p-values for each comparison which were then adjusted using the Benjamini Hochberg method. Data were then transformed logarithmically, using log2(fold-change) and -log10(adjusted p-values), allowing to produce volcano plots (Supplemental file 1). Thresholds of substantial fold-change (log2(fold-change)

greater than 2 or less than -2) and p-value (- log10(p-value) greater than 1.301) were applied to volcano plots to reveal proteins exhibiting robust differential abundance compared between sampling timepoints.

Nascent Proteomics – Data Analyses

Data returned from nascent proteomic experiments were largely handled in Microsoft Excel and was assisted by described processing methodology[15]. For each sample, individual nascent protein abundance was converted to parts per million to normalise for differences in processing and loading, as well as differences in protein synthetic rates and labelling times. The resulting values can be considered to reflect the fraction of the newly synthesised proteome (produced during the labelling period) represented by each protein, with non-detected proteins having an abundance set to 0. A principal components analysis of all samples was performed using the online tool ClustVis[16]. Refined data was uploaded to ClustVis, which returned numerous component values for each sample. For each sample, the first two components (PC1 and PC2) were extracted and plotted to confirm the clustering of replicates. In further analyses, the average abundance calculated from four biological replicates of each sampling timepoint was used. For abundance analyses, the top 100 most abundant nascent proteins from each averaged labelling condition were categorised into modified PseudoCAP (Supplemental file 1). Bar graphs of category occupancy were then produced for each labelling condition. For volcano plots, the fold-changes for each protein were calculated using the average protein abundance for each individual labelling condition. Two tailed paired t-tests were used to calculate p-values for each comparison which were then adjusted using the Benjamini Hochberg method. Data were then transformed logarithmically, using log2(fold-change) and -log10(adjusted p-values), allowing to produce volcano plots (Supplemental file 1). Thresholds of substantial fold-change (log2(fold-change) greater than 2 or less than -2) and p-value (- log10(p-value) greater than 1.301) were applied to volcano plots to reveal proteins exhibiting robust differential expression compared between sampling timepoints. The average abundance of nascent proteins from three key physiological areas were extracted to produce heatmaps. For each nascent protein, the mean abundance retrieved from each individual labelling period was divided by the combined abundance across all three periods. These values of relative abundance were used in heat map production using Microsoft Excel.

Transposon-Insertion Sequencing – Data Analyses

Pooled DNA libraries were prepared for sequencing on the Illumina NextSeq2000 instrument with a P1 reagent kit. Approximately 2.2 - 4.6 million single-end 100 bp reads were obtained per sample. Raw FASTQ files were processed for analysis using the Tn-Seq Pre-Processor (TPP) tool from the Transit

package[17], which finds and filters the transposon sequence from each read and maps the remaining genome sequence to a reference genome using BWA-MEM[18]. Approximately 2.5 - 5.0 million reads per sample were successfully mapped to the UCBPP-PA14 genome (NC_008463.1). Output.sam files were used in conjunction with the genome annotation to summarise counts per gene using the FeatureCounts algorithm of the subread software package[19], disregarding reads that mapped to the first or last 50 bp of each gene because transposon insertion there is less likely to fully disrupt function. Subsequent analyses used a combined dataset from two independent experiments, with each individual sampling timepoint represented by six biological replicates (Supplemental file 1). Replicate 5 from condition A4 (nitrogen starvation transitioned back into nitrogen starvation) and replicate 3 from condition B3 (carbon starvation transitioned into nitrogen starvation) were dropped from further analysis because they were outliers in an MDS plot compared to the other five replicates from these conditions, so these two conditions were represented by only 5 replicates, but these conditions did not feature prominently in our analysis. FeatureCounts data were uploaded to Degust, an online tool for the exploration, analyses and visualisation of large sequencing datasets[20]. Analyses of differential read counts was then performed using a Voom/Limma differential expression method (Supplemental file 1). Benjamini-Hochberg FDR-adjusted p-values for significantly different read counts in any condition are reported in Supplemental File 1. For selected comparisons displayed as volcano plots in Figure 4, FDR-adjusted p-values were calculated for the specific pairwise-comparisons shown. Thresholds of substantial fold-change (log2(fold-change) > 0.585 or < -0.585) and false discovery rate (< 0.05) were applied to returned data to reveal genes exhibiting robust differential read counts between compared samples. These genes were defined as fitness-improving or fitness-diminishing determinants based on the directionality of the calculated fold change value. Genes whose transposon read count reduced in a defined comparison, suggesting that disruption of the gene reduced mutant fitness, were defined as fitness-improving. Genes whose transposon read count increased in a defined comparison, suggesting that disruption of the gene increased mutant fitness, were defined as fitness-diminishing. All substantial and significant fitness-improving and fitness-diminishing determinants returned from select comparisons were functionally categorised into modified PseudoCAP, allowing for bar graphs of category occupancy to be produced (Figure 4F).

Combining Proteomic and Sequencing Datasets

Data retrieved from starvation and transition samples of label-free proteomic and BONCAT proteomic experiments were combined. Only proteins detected in both proteomic datasets were compared, with datasets being re-normalised following the removal of exclusive hits from either (Supplemental file 1). Any protein with an abundance of less than 1 ppm in either dataset was removed before scatter plots

were produced. Proteins belonging to key groups of interest were then highlighted on these scatter plots. Data retrieved from starvation and transition samples/comparisons of BONCAT proteomic and transposon-insertion sequencing experiments were combined. Only proteins/genes detected in both nascent proteomic and transposon insertion sequencing datasets were compared, with the nascent proteomic dataset being re-normalised following the removal of exclusive hits (Supplemental file 1). Scatter plots incorporating both datasets were then produced, with proteins/genes belonging to key groups of interest were then highlighted on these scatter plots.

Experimental Design and Statistical Rationale

In the context of frequently employed starvation experiments, biological replicates were defined as parental LB cultures derived from a distinct colony retrieved from fresh streak plates. For calculations of averages, the mean value of all biological replicates was always used, with the standard deviation of all biological replicates being used as error bars/ribbons. Statistical tests were performed in GraphPad Prism V.10.1.0. Brown-Forsythe/Welch one-way analysis of variance with post-hoc Dunnett's T3 multiple comparison tests were performed to calculate p-values for pairwise comparisons among multiple conditions, timepoints, or strains. In the case of comparisons among strains, each mutant strain was compared only to the wild-type, and multiple comparison corrections were made only for these comparisons. Trendlines were added to longitudinal BONCAT graphs using simple linear regression tools and non-linear fit tools for "binding saturation – one site total". All figures and schematics were produced using a combination of Microsoft Excel, Microsoft PowerPoint, GraphPad Prism V.10.1.0, FlowJo V10.8.1 and Adobe Illustrator.

References:

1 Mathee, K. Forensic investigation into the origin of *Pseudomonas aeruginosa* PA14 - old but not lost. *J Med Microbiol* **67**, 1019–1021, doi:10.1099/jmm.0.000778 (2018).

2 Liberati, N. T. *et al.* An ordered, nonredundant library of *Pseudomonas aeruginosa* strain PA14 transposon insertion mutants. *Proc Natl Acad Sci USA* **103**, 2833–2838, doi:10.1073/pnas.0511100103 (2006).

3 Basta, D. W., Bergkessel, M. & Newman, D. K. Identification of fitness determinants during energy-limited growth arrest in *Pseudomonas aeruginosa*. *mBio* **8**, doi:10.1128/mBio.01170-17 (2017).

4 Shanks, R. M., Caiazza, N. C., Hinsa, S. M., Toutain, C. M. & O'Toole, G. A. *Saccharomyces cerevisiae*-based molecular tool kit for manipulation of genes from gram-negative bacteria. *Appl Environmental Microbiol* **72**, 5027–5036, doi:10.1128/AEM.00682-06 (2006).

5 Choi, K. H. & Schweizer, H. P. mini-Tn7 insertion in bacteria with single attTn7 sites: example *Pseudomonas aeruginosa*. *Nat Protocols* **1**, 153–161, doi:10.1038/nprot.2006.24 (2006).

6 Schlechter, R. O. *et al.* Chromatic Bacteria - A broad host-range plasmid and chromosomal insertion toolbox for fluorescent protein expression in bacteria. *Front Microbiology* **9**, 3052, doi:10.3389/fmicb.2018.03052 (2018).

7 Babin, B. M. *et al.* SutA is a bacterial transcription factor expressed during slow growth in *Pseudomonas aeruginosa*. *Proc Natl Acad Sci USA* **113**, E597–605, doi:10.1073/pnas.1514412113 (2016).

8 Jacobs, M. A. *et al.* Comprehensive transposon mutant library of *Pseudomonas aeruginosa*. *Proc Natl Acad Sci USA* **100**, 14339–14344, doi:10.1073/pnas.2036282100 (2003).

9 Ershov, D. *et al.* TrackMate 7: integrating state-of-the-art segmentation algorithms into tracking pipelines. *Nat Methods* **19**, 829–832, doi:10.1038/s41592-022-01507-1 (2022).

10 Ducret, A., Quardokus, E. M. & Brun, Y. V. MicrobeJ, a tool for high throughput bacterial cell detection and quantitative analysis. *Nat Microbiol* **1**, 16077, doi:10.1038/nmicrobiol.2016.77 (2016).

11 Winsor, G. L. *et al.* *Pseudomonas aeruginosa* Genome Database and PseudoCAP: facilitating community-based, continually updated, genome annotation. *Nucl Acids Res* **33**, D338–343, doi:10.1093/nar/gki047 (2005).

12 Lee, C. R. *et al.* Reciprocal regulation of the autophosphorylation of enzyme I^{Ntr} by glutamine and alpha-ketoglutarate in *Escherichia coli*. *Mol Microbiol* **88**, 473–485, doi:10.1111/mmi.12196 (2013).

13 Pfluger, K. & de Lorenzo, V. Growth-dependent phosphorylation of the PtsN (EIINtr) protein of *Pseudomonas putida*. *J Biol Chem* **282**, 18206–18211, doi:10.1074/jbc.M611110200 (2007).

14 Velazquez, F., Pfluger, K., Cases, I., De Eugenio, L. I. & de Lorenzo, V. The phosphotransferase system formed by PtsP, PtsO, and PtsN proteins controls production of polyhydroxyalkanoates in *Pseudomonas putida*. *J Bacteriol* **189**, 4529–4533, doi:10.1128/JB.00033-07 (2007).

15 Aguilan, J. T., Kulej, K. & Sidoli, S. Guide for protein fold change and p-value calculation for non-experts in proteomics. *Mol Omics* **16**, 573–582, doi:10.1039/d0mo00087f (2020).

16 Metsalu, T. & Vilo, J. ClustVis: a web tool for visualizing clustering of multivariate data using Principal Component Analysis and heatmap. *Nucl Acids Res* **43**, W566–570, doi:10.1093/nar/gkv468 (2015).

17 DeJesus, M. A., Ambadipudi, C., Baker, R., Sassetti, C. & Ioerger, T. R. TRANSIT--A software tool for Himar1 TnSeq analysis. *PLoS Comput Biol* **11**, e1004401, doi:10.1371/journal.pcbi.1004401 (2015).

18 Li, H. & Durbin, R. Fast and accurate short read alignment with Burrows-Wheeler transform. *Bioinformatics* **25**, 1754–1760, doi:10.1093/bioinformatics/btp324 (2009).

19 Liao, Y., Smyth, G. K. & Shi, W. featureCounts: an efficient general purpose program for assigning sequence reads to genomic features. *Bioinformatics* **30**, 923–930, doi:10.1093/bioinformatics/btt656 (2014).

20 Powell, D. Degust: interactive RNA-seq analysis. doi:10.5281/zenodo.3258932 (2019).
